# Recent Progresses on Hybrid Lithium Niobate External Cavity Semiconductor Lasers

**DOI:** 10.3390/ma17184453

**Published:** 2024-09-11

**Authors:** Min Wang, Zhiwei Fang, Haisu Zhang, Jintian Lin, Junxia Zhou, Ting Huang, Yiran Zhu, Chuntao Li, Shupeng Yu, Botao Fu, Lingling Qiao, Ya Cheng

**Affiliations:** 1 The Extreme Optoelectromechanics Laboratory (XXL), School of Physics and Electronic Science, East China Normal University, Shanghai 200241, China; zwfang@phy.ecnu.edu.cn (Z.F.); hszhang@phy.ecnu.edu.cn (H.Z.); jxzhou@phy.ecnu.edu.cn (J.Z.); ht19930312@163.com (T.H.); 52270920003@stu.ecnu.edu.cn (Y.Z.); 52270920001@stu.ecnu.edu.cn (C.L.); ya.cheng@siom.ac.cn (Y.C.); 2 Engineering Research Center for Nanophotonics and Advanced Instrument, School of Physics and Electronic Science, East China Normal University, Shanghai 200241, China; 3 State Key Laboratory of High Field Laser Physics and CAS Center for Excellence in Ultra-Intense Laser Science, Shanghai Institute of Optics and Fine Mechanics (SIOM), Chinese Academy of Sciences (CAS), Shanghai 201800, China; jintianlin@siom.ac.cn (J.L.); spyu@siom.ac.cn (S.Y.); fubt@shanghaitech.edu.cn (B.F.); qiaolingling@siom.ac.cn (L.Q.); 4 Center of Materials Science and Optoelectronics Engineering, University of Chinese Academy of Sciences, Beijing 100049, China; 5 State Key Laboratory of Precision Spectroscopy, East China Normal University, Shanghai 200062, China; 6 School of Physical Science and Technology, ShanghaiTech University, Shanghai 200031, China; 7 Collaborative Innovation Center of Extreme Optics, Shanxi University, Taiyuan 030006, China; 8 Collaborative Innovation Center of Light Manipulations and Applications, Shandong Normal University, Jinan 250358, China; 9 Shanghai Research Center for Quantum Sciences, Shanghai 201315, China; 10 Hefei National Laboratory, Shanghai 230088, China

**Keywords:** thin film lithium niobate, photonic integrated circuit, external cavity semiconductor laser, microresonator, photolithography-assisted chemo-mechanical etching (PLACE), butt coupling, hybrid integration, narrow linewidth

## Abstract

Thin film lithium niobate (TFLN) has become a promising material platform for large scale photonic integrated circuits (PICs). As an indispensable component in PICs, on-chip electrically tunable narrow-linewidth lasers have attracted widespread attention in recent years due to their significant applications in high-speed optical communication, coherent detection, precision metrology, laser cooling, coherent transmission systems, light detection and ranging (LiDAR). However, research on electrically driven, high-power, and narrow-linewidth laser sources on TFLN platforms is still in its infancy. This review summarizes the recent progress on the narrow-linewidth compact laser sources boosted by hybrid TFLN/III-V semiconductor integration techniques, which will offer an alternative solution for on-chip high performance lasers for the future TFLN PIC industry and cutting-edge sciences. The review begins with a brief introduction of the current status of compact external cavity semiconductor lasers (ECSLs) and recently developed TFLN photonics. The following section presents various ECSLs based on TFLN photonic chips with different photonic structures to construct external cavity for on-chip optical feedback. Some conclusions and future perspectives are provided.

## 1. Introduction

Compact lasers with narrow linewidth, wide spectral tunability, high coherence, and low frequency/phase noise are the fundamental building blocks for developing chip-scale technology in high-speed optical communication [1,2,3], coherent detection [4], precision metrology [5,6], laser cooling [7,8], coherent transmission systems [9], light detection and ranging (LiDAR) [10,11,12], and so on. Currently, the most common solutions for realizing narrow-linewidth lasers are constructed based on solid-state, fiber, and semiconductor lasers owing to the high power efficiency and easy maintenance [13]. The solid-state lasers and fiber lasers possess relatively longer resonant cavity lengths, which means longer photon lifetimes and therefore it is easy to achieve good phase/frequency noise performance. However, both solid-state and fiber lasers inevitably suffer from larger sizes and weights, and the manufacturing and packaging costs are also very high [14]. Currently, great efforts have been devoted to developing narrow-linewidth semiconductor lasers due to the chip-level dimensions, capability of directly electrically pumping and low manufacturing cost. Distributed-feedback (DFB) [15,16] and distributed Bragg reflector (DBR) [17] lasers with a linewidth in the order of a hundred kHz have been realized by improving the grating coupling efficiency and cavity length. Nevertheless, the finite cavity length defined by the chip footprint hinders further reduction in the linewidth [18,19,20]. Meanwhile, fabrication processes such as secondary epitaxy and precise lithography for achieving narrow linewidth raise the device intricacy and cost. Therefore, narrow-linewidth semiconductor lasers are typically coupled with mode selection devices outside the cavity to achieve frequency filtering and linewidth suppression [21,22], which is called “external cavity diode lasers (ECDLs)” or “external cavity semiconductor lasers (ECSLs)”. Standard reflective diffraction gratings, such as holographic or blazed gratings, have been successfully employed as the frequency selectors in the ECSL systems [22,23,24]. Due to the presence of the discrete bulk optical components and the fact that the grating or reflective mirror is adjusted mechanically, the overall integrity of the laser system is poor, making it sensitive to external vibrations. The laser wavelength is also prone to mode hopping due to external vibrations and changes in ambient temperature, which requires additional compensation modules. Fiber Bragg gratings [25] and crystalline whispering gallery mode (WGM) resonators [14,26] have also been reported to replace some of the bulk components, but lenses and prisms are still inevitably used for coupling the light in and out of the gain chips and the external cavity elements. The configuration of these ECSLs impedes further improvement in the device size, tuning rate, and packaging cost.

With the development of photonics integration technology, waveguide external cavity structures have emerged as an alternative solution to realize high-performance ECSL [13,27,28,29,30]. By utilizing waveguide external cavities to achieve narrow linewidth, the size and volume of the system are greatly reduced. Furthermore, these structures can be integrated with other components, enhancing the flexibility and reliability of the system. Several miniaturized ECSLs have been assembled by integrating silicon-based semiconductor photonic chips with semiconductor gain chips. Photonic integrated circuits (PICs) with Vernier microring resonators (MRR) [31,32,33,34,35,36], distributed Bragg reflectors, Sagnac loop reflectors (SLRs) [35,36,37], and Mach–Zehnder interferometers [33,38] have been fabricated on silicon (Si) [31,39,40], silicon dioxide (SiO_2_) [36], and silicon nitride (Si_3_N_4_) [33,34,35] and other material platforms to form a laser cavity. The photonic chip integrated ECSL features high reliability and low power consumption, as well as ultra-narrow linewidth and a wide tuning range for the external cavity structure.

Recently, thin film lithium niobate (TFLN) has drawn tremendous attention as a prospective photonic substrate because of its superior optical properties. As a traditional optical crystal, lithium niobate possesses a broad transparency window, high nonlinear optical coefficients, and rapid electro-optic tuning [41,42,43,44,45,46]. It can even serve as gain medium itself via rare-earth ion doping [47,48]. Thanks to the breakthroughs in the TFLN photonic microstructuring technology, a large amount of excellent passive and active photonic devices [49] have already been demonstrated, such as modulators [50,51,52], frequency converters [53,54], beam splitters [55,56], delay lines [57], microlasers [58,59,60,61] and waveguide amplifiers [62,63,64,65,66,67]. The TFLN PIC technology has proven its capabilities in achieving high optical and electro-optical performance (i.e., losses, spectral and phase precision, etc.), close to that of bulk optical devices, whilst offering scalability in terms of the integration level as well as energy efficiency benefited by lithographic fabrication, especially the photolithography-assisted chemo-mechanical etching (PLACE) fabrication technique [68,69,70]. Tunable and reconfigurable PIC applications are becoming practical, ranging from microwave-to-optical wave convertors [71,72] and detectors [73,74,75,76,77] to spectrometers [78,79,80], combs [81,82,83], neural network computer [84], ultrafast lasers [85,86], and telecom [87,88,89,90] devices.

As an indispensable part of the large-scale PIC applications, the TFLN platform is eager for the development of on-chip electronically pumped optical sources. However, due to the intrinsic material properties of lithium niobate, it is difficult to directly fabricate lasers on pure TFLN wafer. On-chip microlasers have been demonstrated on rare-earth ion (REI)-doped TFLN wafers, which are no longer as suitable as the single crystalline TFLN wafer to support ultra-low transmission loss and inevitably need external pump laser sources. Heterogeneous integration of III–V semiconductor lasers on a TFLN platform is reported to be realized by micro-transfer printing (μTP) [91] and wafer bonding [92], but suffers from fabrication complexity caused by the process compatibility issues of the two different materials and limited output lasing power. Hybrid integration of III–V semiconductor lasers with TFLN platform allows the combination of nearly mature III–V semiconductor end products with high-performance TFLN photonic chips without considering their process compatibility [93]. Thus, despite the challenges like output power and coupling loss it currently encounters, great effort has been dedicated to developing hybrid ECSL with TFLN PICs.

This review focuses on the recent progress in hybrid lithium niobate external cavity semiconductor lasers. In Section 2, we review the latest hybrid ECSLs integrated with different TFLN photonic structures. In Section 3, we give a brief summary and outlook on this rapidly developing field of research.

## 2. Hybrid ECSLs Integrated with Different TFLN Photonic Structures

According to the modified Schawlow–Townes linewidth formula [94,95], the full width at half maximum (FWHM) linewidth of the semiconductor laser can be written as

(1) $$\Delta \nu =\frac{h\nu v_{g}^{2}\left(\alpha_{i}+\alpha_{m}\right)\alpha_{m}n_{sp}}{8\pi P_{out}}\left(1+\alpha_{H}^{2}\right)$$

(2) $$\alpha_{m}=\frac{1}{2L}ln(\frac{1}{R_{1}R_{2}})$$

where hν represents the photon energy, $\alpha_{i}$ and $\alpha_{m}$ are the internal loss of the cavity and the optical loss induced by the interface, $v_{g}$ is the group velocity of the light, $n_{sp}$ is the spontaneous emission factor, $\alpha_{H}$ is the linewidth enhancement factor, $P_{out}$ is the output power at the facet, *L* is the cavity length, $R_{1}$ and $R_{2}$ is the reflectivity of the rear (front) facet. Therefore, increasing the cavity length and improving the power are effective methods to compress the laser linewidth. However, the increase in the cavity length results in a decrease in the spectral interval between the adjacent resonance modes, which is much smaller than the gain bandwidth of the semiconductor laser diode, making it difficult to achieve stable single longitudinal mode output. Thus, it is also necessary to insert mode selection components and suppress side modes when constructing external cavity lasers. In 1980, R. Lang and K. Kobayashi experimentally observed a single-mode laser based on external optical feedback effects [21]. It is now a common technique to acquire linewidth compression via external cavities. ECSLs can be divided into two parts; namely, an active internal cavity that provides gain and a passive external cavity that provides optical feedback. Light emitted from the active gain medium (usually from a laser diode or an optical amplifier (SOA) chip) is fed back after passing through a low-loss passive external medium, and the introduction of a low-loss passive external cavity increases the photon lifetime of the laser system, thereby narrowing the linewidth. In this section, we will introduce several ECSLs based on the TFLN PIC external cavity with different photonic configurations.

### 2.1. ECSLs Intergrated with TFLN Microrings

High-Q TFLN microring resonators with monolithically coupled bus waveguides are one of the simplest structures to form an external cavity. The ring-shaped waveguide resonator supports WGM with high Q factors. The material or fabrication inhomogeneities on the interface of the LN ring and the surroundings leads to the resonant Rayleigh scattering effect. Thus, a portion of incoming radiation with the eigenfrequencies of WGMs feedbacks to the cavity, and triggers the self-injection locking effect resulting in a significant reduction in the laser linewidth [96]. Under the self-injection locking condition, the semiconductor laser runs at the resonant frequency of the external microring cavity. The frequency noise suppression ratio is

(3) $$\frac{\delta \omega }{\delta \omega_{free}}\approx \frac{Q_{d}^{2}}{Q^{2}}\times \frac{1}{16\Gamma_{m}^{2}\left(1+\alpha_{g}^{2}\right)}$$

where *δω* represents the linewidth of the self-injection-locked semiconductor laser, *δω_free_* signifies the linewidth of the free-running DFB laser, *Q_d_* and *Q* denote the quality factors of the laser diode cavity and the LN microring cavity, respectively, and *α_g_* represents the phase–amplitude coupling factor. Therefore, the loaded *Q* factors of the ring resonators, which are relevant to the fabrication technique and the device design, are important to the performance for the device.

**Figure 1:**
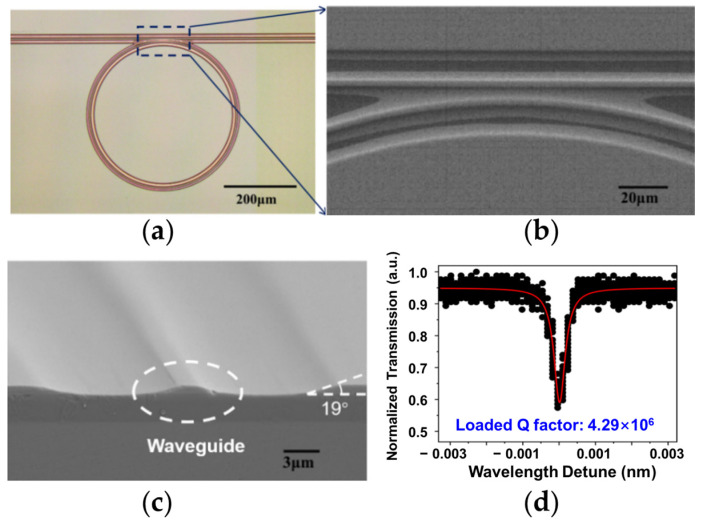
High Q thin film lithium niobate (TFLN) microring side-coupled with a ridge waveguide fabricated by PLACE technique [107]. (**a**) Optical microscope image of the photonic structure. (**b**) The scanning electron microscope (SEM) image of the coupling region between the microring resonator and the straight waveguide, which is shown by the blue dotted line box in (**a**). (**c**) The SEM image of the cross section of the strip waveguide. (**d**) Transmission spectra after annealing. The red Lorentz fitting curve indicates a loaded Q factor of 4.29 × 10^6^. Reprinted with permission from [107] © Optical Society of America.

In the past few years, several solutions for low-loss TFLN photonic fabrication have been developed successively, for example, femtosecond laser assisted focused ion beam milling [97,98], ultraviolet (UV) lithography followed by plasma dry etching [99,100], electron beam lithography (EBL) followed by Ar ion milling [101] and the photolithography-assisted chemo-mechanical etching (PLACE) [70] technique. For TFLN microrings coupled with waveguides fabricated by ion milling or dry etching, the typical Q factors are about 10^4^~10^5^. The PLACE technique has been used to etch the TFLN, leaving an ultra-smooth sidewall, which enables ultra-low loss meter-scale waveguides [57], ultra-high Q microrings [102], and free-standing microdisks [103]. Due to the limited etch ratio between the chromium hard mask and lithium niobate, the overlap between the microring and the bus waveguides leads to a gradual saddle-shaped cross section of the coupling area. Efforts have been devoted to optimizing the coupling condition and the loaded Q factor by improving the fabrication processes and the waveguide geometry [104,105,106]. Microrings composed by connecting quarter Bezier curve waveguides have been proved to achieve better mode-coupling conditions compared to the perfect circle-shaped waveguide resonator [105]. And it is also effective to change the waveguide cross section design and take an additional post-anneal process, as shown in Figure 1a–c [107]. The highest Q factor of a monolithic microring side-coupled with a rib waveguide reaches 4.29 × 10^6^, as shown in Figure 1d. And the intrinsic Q factor was determined as high as 4.04 × 10^7^, corresponding to a propagation loss of 0.0091 dB cm^−1^ [69,108,109,110]. Table 1 shows the measured loaded Q factors of 12 microring samples prepared in the same PLACE batch without annealing. There are 11 samples existing resonant mode with a loaded Q factor higher than 10^6^, indicating a process stability of more than 91.6%.

**Figure 2:**
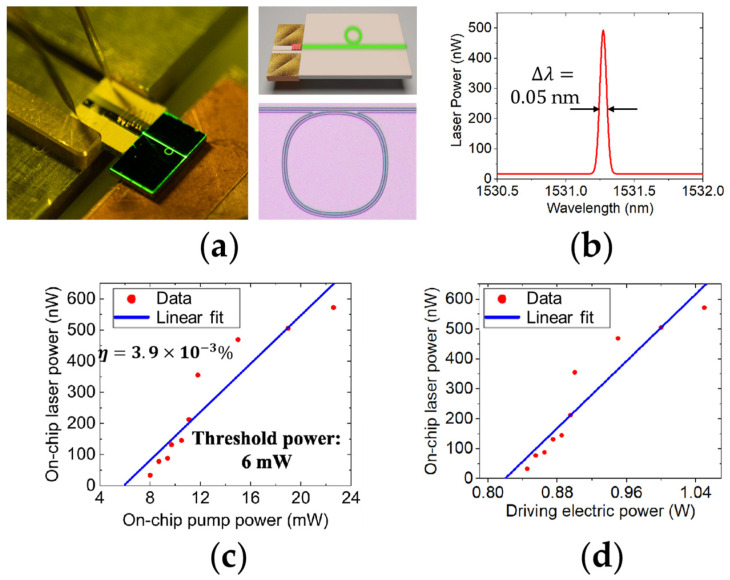
Compact hybrid Er^3+^-doped lithium niobate microring laser [111]. (**a**) Picture of the device. Upper right inset: Illustration of the device. Bottom right inset: the Er^3+^-doped lithium niobate microring captured by optical microscope. (**b**) The lasing spectrum collected from the bus waveguide. (**c**) The on-chip lasing power of the microring laser as a function of the increasing input pump power. (**d**) The on-chip lasing power of the microring laser as a function of the increasing driving electric power. Reprinted with permission from [111] © Optical Society of America.

In 2022, J. Zhou, et al. demonstrated a compact hybrid lithium niobate microring laser [111]. It is composed of a 980 nm pump laser diode and an Er^3+^-doped TFLN microring, as shown in Figure 2a. The upper right inset of Figure 2a is the illustration of the compact hybrid laser on TFLN integrated platform. The pump laser diode of a center wavelength of 976 nm has a linewidth of 2.18 nm and a footprint of 4 μm × 1 μm. The microring is fabricated by PLACE technology on a 500 nm Z-cut Er^3+^-doped TFLN. In order to improve the Q value of the microring, a four-segment Bezier curve is used instead of the traditional arc curve, and the bending radius of the microring is 200 μm (as presented in the bottom right inset of Figure 2a). The Er^3+^-doped lithium niobate microring produces a single-mode laser in the C-band under the excitation of the 980 nm diode-pumped light source, with a center wavelength of 1531 nm and a linewidth of 0.05 nm as shown in Figure 2b. The relationship between on-chip laser power and on-chip pump power is plotted in Figure 2c, where the threshold power of the pump laser is 6 mW and the conversion efficiency is calculated to be 3.9 × 10^−3^%. Figure 2d shows the on-chip laser power as a function of the driving electric power and that the threshold current is 0.5 A when the operating voltage is 1.64 V. Although this chip-based laser is not strictly an ECSL, it has stimulated further research on the integration of semiconductor diode with lithium niobate photonics chips.

**Figure 3:**
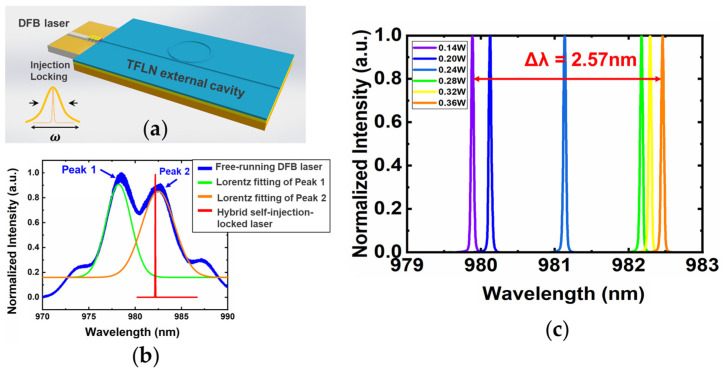
Compact hybrid self-injection-locked lithium niobate microring laser [112]. (**a**) Illustration of the narrow-linewidth laser. (**b**) Comparison of the laser linewidth for the free-running DFB laser (blue curve) and the self-injection-locked microlaser (red curve). The green and orange curve are the Lorentz fitting lines of the double lasing peak of the free-running DFB laser. (**c**) The lasing wavelength drifts with the increasing electrical pumping power.

A self-injection locking narrow-linewidth integrated laser [112] has been demonstrated by butt coupling a DFB diode with a LN chip. Figure 3a illustrates the configuration design of the narrow-linewidth self-injected locking laser, comprising a commercially available 980 nm CoS laser diode and a LN microring monolithically coupled with a straight bus waveguide. The Q factor of the microring is 6.91 × 10^5^. Figure 3b presents the emission spectra of the free-running DFB laser diode and the hybrid integrated microlaser. The blue curve represents the measured laser emission from the free-running DFB laser. Two lasing peaks with a central wavelength of 978.79 nm and 982.47 nm are plotted in green and orange with a linewidth of 2~3 nm. So, the Q factors of the free running DFB laser are the order of ~10^2^. It is clear that when the external LN ring cavity is coupled with the DFB laser diode, the lasing behavior transit from multimode to single mode occurs under the self-injection locking condition. The hybrid laser device is compelled to oscillate at the resonant wavelength of the LN microring around 982 nm, providing a narrow-linewidth lasing spectrum as the red curve shown in Figure 3b. The linewidth is 35 pm limited by the resolution of the optical spectral analyzer. More accurate linewidth measurement can be conducted later by using the self-delayed heterodyne method. The wavelength tuning is performed by increasing the applied electrical power of the DFB laser as shown in Figure 3c. The lasing wavelength experiences a nonlinear redshift as the electrical pump power varies from 0.14 W to 0.36 W. The tuning range covers a wavelength band of 2.57 nm. Continuous wavelength tuning can be realized by through the integration of microelectrodes close to the LN microring resonator.

**Figure 4:**
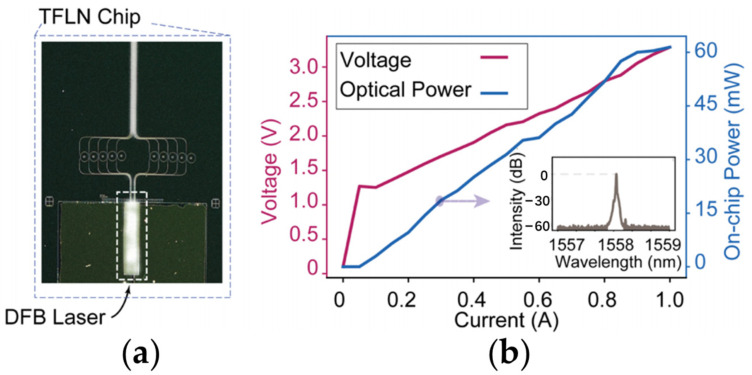
Characterization of the monolithically integrated high-power III-V/TFLN laser [87]: (**a**) The microscope image of a DFB laser flip-chip bonded with a TFLN chip, which includes multiple waveguides that are coupled to microring resonators. (**b**) The light–current–voltage (LIV) measurement of the device. The inset: the lasing spectrum of the device. Reprinted with permission from [87] © Optical Society of America.

The DFB laser–TFLN cavity coupling structure is also employed to high-power hybrid integrated laser and transmitter [87]. The InP DFB laser is monolithically integrated with a pre-fabricated TFLN chip of passive microring arrays by flip-chip bonding, as pictured in Figure 4a. Optimization of the mode profile overlap between the eigenmodes of TFLN waveguide and the DFB laser is performed to obtain an on-chip optical power of 60 mW when the pump current is set to 1.0 A, as indicated in Figure 4b. The inset of Figure 4b reveals that the integrated laser is under the single-mode operation without mode hopping. The linewidth is estimated to be lower than 1 MHz via a delayed self-heterodyne technique. It is noteworthy that the resonance condition of DFB laser diode and the TFLN microring is not satisfied in this case. An electrically pumped high-power transmitter with an electro-optic (EO) bandwidth over 50 GHz is also realized by flip-chip bonding a DFB laser with a TFLN Mach–Zehnder interferometer modulator.

**Figure 5:**
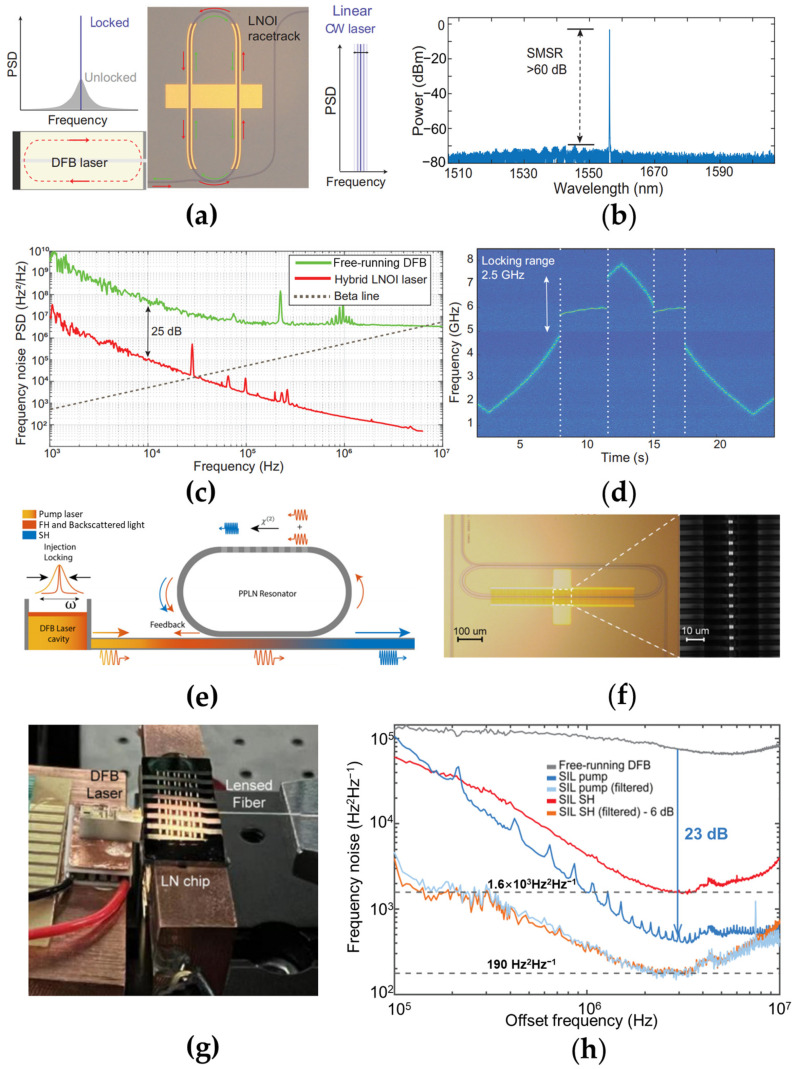
Low-noise III-V/TFLN self-injection-locked laser using LN racetrack external cavity [113,114]. (**a**) Schematic of III-V/TFLN butt-coupling laser [113]. (**b**) Optical emission spectrum of the device. (**c**) The frequency noise spectra of the hybrid laser (red) and the free-running DFB laser diode (green). (**d**) The lasing frequency of the self-injection-locked hybrid laser versus the diode current. The mode locked area is marked by the white dashed lines, which indicate a locking range of about 2.5 GHz. (**e**) Schematic of the III-V/TFLN hybrid frequency conversion laser [114]. The periodically poled lithium niobate (PPLN) racetrack resonator provides not only the backscattering portion of the lightwaves for the self-injection locking but also the quasi-phase matching for the second-harmonic generation (SHG) process. (**f**) Optical microscope image of the lithium niobate photonic integrated chip with a PPLN racetrack resonator and the uniform period poling structure captured by a confocal microscope. (**g**) The picture of the frequency conversion laser system. (**h**) The frequency noise spectra of the free-running DFB laser (gray), self-injection locking pump light (blue), and self-injection locking SH signal (red), respectively.

Z. Li, et al. demonstrate a low noise III-V/TFLN hybrid laser by triggering self-injection locking with a TFLN racetrack external cavity [113]. As shown in Figure 5a, the straight waveguide section of the racetrack microring structure is easy to integrate with microelectrodes for electric field distribution engineering. The side mode suppression ratio (SMSR) is over 60 dB, as plotted in Figure 5b. The frequency noise of the hybrid laser, labeled by red solid line in Figure 5c, is 52 Hz^2^ Hz^−1^ at 6 MHz offset, which is reduced by more than 25 dB over the whole spectrum compared to that of the free-running DFB laser diode (green solid line). The FWHM linewidth is measured to be 242 kHz at 1 ms integration time. A locking range as wide as 2.5 GHz is achieved by the optical feedback of the TFLN racetrack resonator, as shown in Figure 5d. A reduction in the laser linewidth and the lasing frequency fluctuation with the diode current is clearly observed.

A multimode add–drop race-track microring with a multimode interferometric coupler (MMRA-MRR) structure on a TFLN chip is also reported to serve as an external cavity [115]. The multimode waveguide and the add–drop structure enable the device with a low transmission and a high coupling efficiency, along with additional injection feedback. Thus, a narrowed linewidth of 2.5 kHz is obtained.

Recently, a narrow-linewidth frequency conversion laser which generates high-coherence second harmonic lightwaves [114] was demonstrated. It consists of a DFB diode pump laser butt-coupled with a partially period-poled lithium niobate (PPLN) racetrack resonator, whose straight PPLN waveguide section is produced to achieve the quasi-phase match for the on-chip second harmonic generation (SHG). The conceptional illustration and the pictures of the device are as shown in Figure 5e–g. The length of the PPLN waveguide section is about 630 μm, and the poling period is determined to be 4.3 μm for satisfying the double resonance of the pump and SH wavelength. The total cavity length is 1.9 mm, thus the free-spectral range (FSR) around the wavelength of ~1560 nm is 0.584 nm. Figure 5h shows that the hybrid laser features a low frequency noise for both pump wavelength and the SH wavelength. Compared to the free-running DFB laser, the high-offset-frequency pump noise is significantly reduced by ~23 dB due to the effect of the self-injection-locking process. The SHG noise reaches a level of 760 Hz^2^ Hz^−1^(=4 × 190 Hz^2^ Hz^−1^). The SHG noise reaches a level of 1600 Hz^2^ Hz^−1^ at around 3 MHz offset frequency. A near-visible SHG output near 780 nm is achieved with a laser linewidth as narrow as 4.7 kHz and an on-chip converted power over 2 mW. It paves the way to achieve affordable production of high-coherence visible microlasers for high precision sensing/navigation/timing applications.

### 2.2. ECSL Integrated with TFLN Loop Mirror and DBR

**Figure 6:**
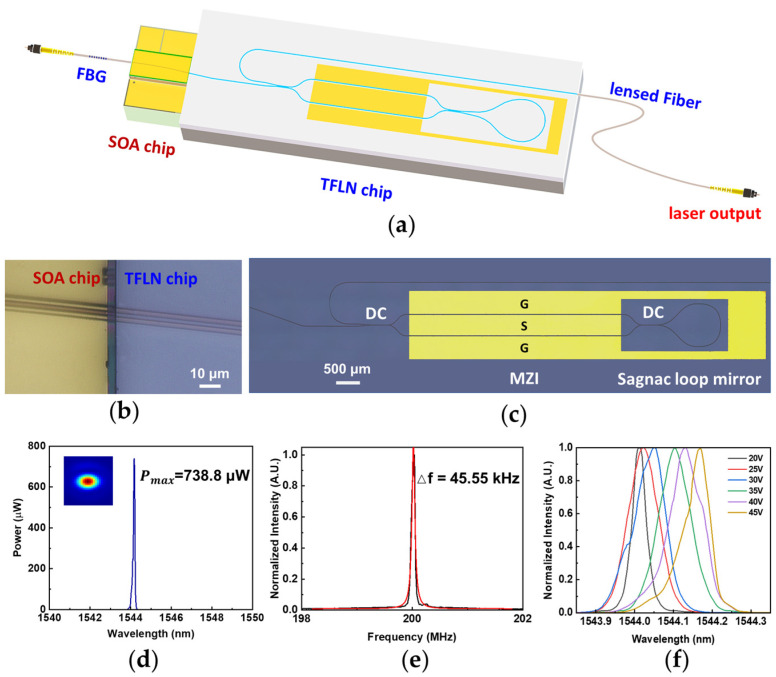
Electro-optically tunable narrow-linewidth laser based on TFLN loop mirror [118]. (**a**) Schematic view of the device. (**b**) The microscope image of the butt-coupling area between the TFLN and SOA chip. (**c**) The top view image of the TFLN chip captured by the optical microscope. (**d**) The emission spectrum of the microlaser. It operates under the single mode with a maximum output power of 738.8 μW. Inset: The optical field distribution of the laser output captured by the infrared camera. (**e**) The laser linewidth is measured to be 45.55 kHz. (**f**) The lasing peak shifts along with the increasing applied voltages. Reprinted from [118] with permission from AAAS.

Diode lasers with Fabry–Pérot (FP) cavities formed by Bragg gratings or high refractive coatings have been proved to produce high output power. The limited cavity length and sophisticated processing technique obstructs the further improvement of the lasing performance. Utilizing external monolithically integrated reflective mirrors has become an effective solution to extend the cavity length. One of the ideal on-chip reflectors for building an external FP cavity laser is the Sagnac loop reflector (SLR). It is actually a 2 × 2 directional coupler (DC) with one side of the waveguides connected together as a loop. The mirror reflectivity of the SLR is determined by the coupling ratio, which plays an important role in the spectral fineness of the integrated laser. Recently, a single-mode SLR FP microlaser was reported on a TFLN wafer [116,117]. Furthermore, an electro-optic tunable narrow-linewidth laser is also realized by butt coupling a SOA chip to the end of a TFLN photonic chip [118]. As shown in Figure 6a, the FP laser cavity is constructed using a fiber Bragg grating and a TFLN SLR with a Mach-Zehnder interferometer (MZI) to select the lasing wavelength. Figure 6b is the optical microscope image of the end coupling area. Figure 6c is the top view image of the TFLN chip. In addition, the patterns of the ground–signal–ground (GSG) electrodes on the TFLN chip are also obtained through selective direct writing using femtosecond laser.

The III-V/TFLN hybrid integrated FP cavity contributes to achieve single spatial and spectral mode, narrow linewidth, and fast on-chip frequency tuning simultaneously. The output power of the integrated laser is 738.8 μW when the applied voltage on the GSG electrode is tuned to 45 V. The intrinsic linewidth of 45.55 kHz is obtained at the C-band as shown in Figure 6d,e. Figure 6f shows a dependence of the lasing wavelength on the applied voltage of the MZI, indicating a frequency tuning range of 20 GHz. The laser is unique in its hybrid external cavity design which combines a TFLN tunable SLR and an FBG, both of which can be easily integrated to the SOA chip. The combination of the two makes the filtering of a single frequency out of the laser cavity straightforward and allows for high-precision wavelength tuning thanks to the highly reflective Sagnac loop reflector.

**Figure 7:**
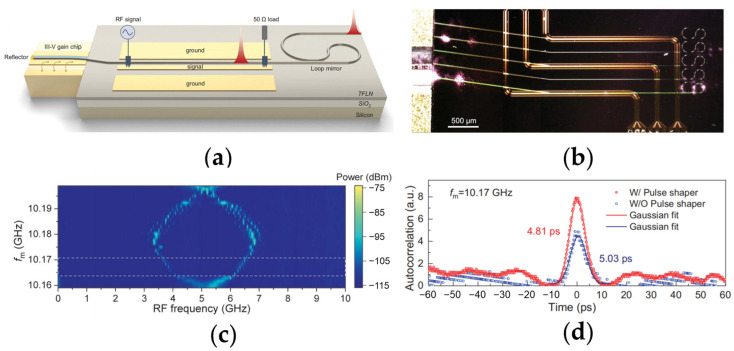
Integrated TFLN ultrafast mode-locked laser (MLL) [86]. The schematic (**a**) and the top view image taken by the microscope (**b**) of the hybrid MLL. (**c**) The device operates in mode locked condition when the applied radio frequency (RF) is from 10.165 GHz to 10.173 GHz. (**d**) The Gaussian fitting curve of the intensity autocorrelation data of the MLL output shows the generation of an ultrafast pulse with a pulse width of 4.81 ps and a repetition rate of 10.17 GHz.

Similar structure can also be utilized to obtain an ultrafast, actively mode-locked laser (MLL) [86]. As illustrated in Figure 7a,b, the device consists of an electrically pumped single-angled facet (SAF) gain chip and a TFLN chip with a EO tunable phase modulator (PM) followed by an SLR. The device can operate in the mode-locked regime when a certain radio frequency (RF) signal is employed on the GSG electrodes of the PM. The mode-locked range is from 10.165 GHz to 10.173 GHz, as labeled in Figure 7c by the white dashed box. The red Gaussian fitting curve of the intensity autocorrelation trace in Figure 7d indicates that the MLL emits ultrafast pulses with a pulse width of 4.8 ps at a repetition rate of ~10 GHz.

**Figure 8:**
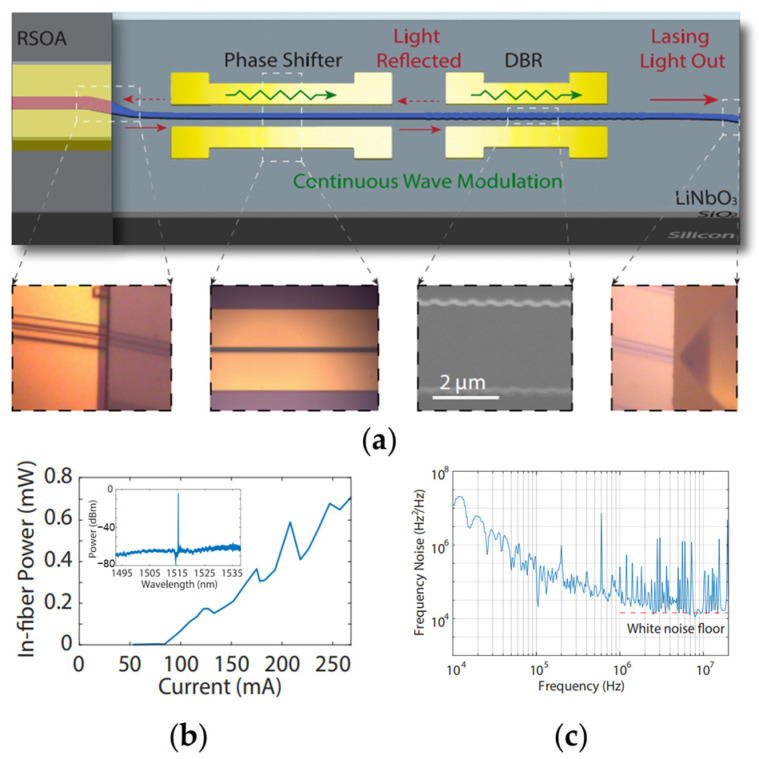
Integrated TFLN distributed Bragg reflector (DBR) based tunable ECSL device [119]. (**a**) The schematic of the tunable ECSL device. Insets: the optical images or the SEM image of the device. (**b**) The LI curve of the laser. Inset: the lasing spectrum obtained by an optical signal analyzer showing that the laser operates in the single mode. (**c**) The frequency noise is measured by analyzing the phase noise captured by a real-time oscilloscope. A white noise floor of ~1.5 × 10^4^ Hz^2^/Hz is marked by the red dashed line, indicating an intrinsic linewidth of 94 kHz.

Another approach to achieve on-chip optical feedback is by utilizing a waveguide with periodic grooves, which functions as a DBR [119]. Figure 8a shows the schematic of a DBR-based tunable ECSL device. The gain part is a III-V reflective SOA chip, while the external TFLN chip consists of a phase shifter (PS), a DBR structure and a bending output-coupling waveguide. The recorded optical spectrum of the laser shown in the inset of Figure 8b clearly indicates single-mode lasing at 1516.21 nm with a SMSR of over 50 dB. As shown in Figure 8b, the laser exhibits a lasing threshold of 80 mA. A laser power of 0.7 mW is recorded in fiber at a pumping current of 250 mA. The frequency noise of the laser is shown in Figure 8c, measured by the correlated delayed self-heterodyne method. The white frequency noise floor of ~1.5 × 10^4^ Hz^2^/Hz is observed at the offset frequency around 8 MHz, which corresponds to an intrinsic linewidth of 94 kHz.

### 2.3. ECSL Integrated with Compound TFLN PICs

**Figure 9:**
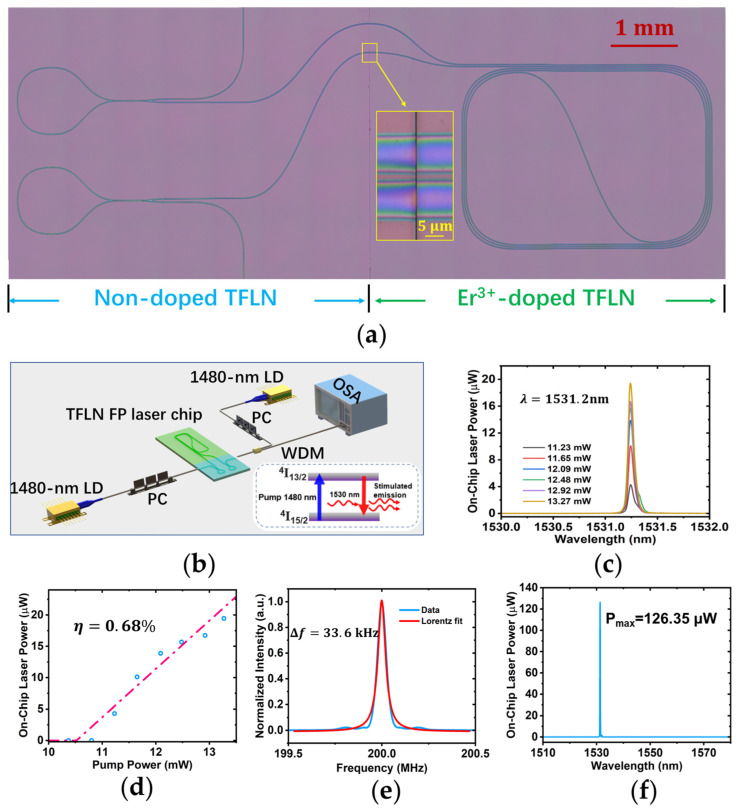
Active/passive integrated TFLN FP cavity laser [121]. (**a**) The top view microscope image of the TFLN chip. Inset: The low loss interface of the active and passive TFLN waveguides marked by the yellow square. (**b**) The schematic of the experiment for characterizing the lasing behavior of the device. Inset: the schematic of the Er^3+^ ion energy level and the stimulated emission triggered by the 1480 nm pump light. (**c**) The emission spectra of the device at different pumping powers. (**d**) The on-chip power of the FP laser along with the increasing pump power. (**e**) A laser linewidth of 33.6 kHz is measured via the linewidth of the beating signal collected from the delayed self-heterodyne interferometer. (**f**) The laser spectrum features multi-mode peaks at a pumping power of 34 mW. Reprinted from [121] with permission from Elsevier.

Various active photonic devices, such as narrow-linewidth microlasers, waveguide amplifiers, and quantum emitters, have been demonstrated these days on rare-earth ion (REI)-doped TFLN wafers. In order to scale up the high performance photonic chips, a robust low-loss optical interface for passive/active photonic integration has also been accomplished [120]. Such a compound TFLN PIC can function not only as an external mode-selection element but also as a gain chip, which is proved to be suitable for constructing integrated ECSL [121]. The hybrid laser chip fabricated by the PLACE technique contains a spiral waveguide on the Er^3+^-doped TFLN section and two SLRs on the non-doped TFLN section, as shown in Figure 9a. The footprint of the TFLN chip is only 10 mm × 4 mm. The spiral waveguide with a total length of 5 cm serves as an on-chip optical amplifier which absorbs the pump light at the wavelength of 1480 nm and provides gain signal at the wavelength of 1530 nm as illustrated in the inset of Figure 9b. The DC coupling efficiency of the two SLRs for the gain signal and the pump light is adjusted to be 44% and 30.6%, respectively, by optimizing the DC gap and coupling length.

Characterization of the device is conducted using the experimental setup shown in Figure 9b. The lasing spectra at different pumping powers ranging from 11.23 mW to 13.27 mW are plotted in Figure 9c; manifesting of the passive/active integrated laser operates in a single-mode regime around the central wavelength of ~1531 nm. The negligible drift of the central wavelength at increasing pump powers indicates that the hybrid laser effectively suppresses the photorefractive and thermo-optic effects. The lasing power trace along with the increasing pump power is depicted in Figure 9d. The lasing threshold is derived from the linear fitting curve, which is estimated to be 10.5 mW. The lasing efficiency is obtained by the slope as 0.68%, which surpasses that of the Er^3+^-doped TFLN lasers demonstrated so far. The linewidth of the passive/active monolithically hybrid laser is examined utilizing the optical delayed self-heterodyne interferometer measurement. The beating signal detected by the fast photo detector is recorded as shown in Figure 9e, following a Lorentz fitting curve with a central frequency of 200 MHz and a retrieved linewidth of 67.2 kHz. Thus, the laser linewidth is measured to be 33.6 kHz. It is observed that the output spectrum of the passive/active TFLN FP laser features multi-mode lasing with a maximum output of 126 μW when the pumping power of the laser diode (LD) is 34 mW, as shown in Figure 9f. This multi-mode lasing behavior is restrainable by inserting frequency selective components into the passive/active TFLN PIC, for example, microring filters or Bragg grating waveguides.

**Figure 10:**
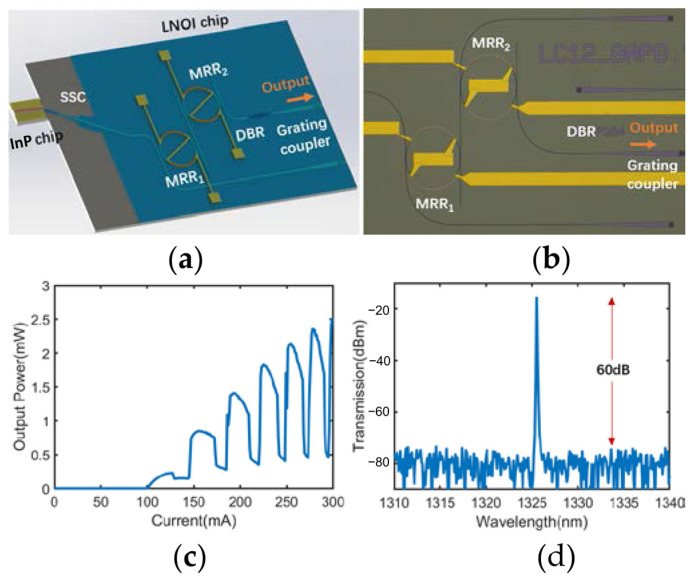
Hybrid O-band TFLN/III-V integrated tunable microlaser [122]. (**a**) Schematic of the laser. (**b**) Optical microscope image of the integrated TFLN chip. (**c**) Continuous-wave L–I curve of the microlaser. (**d**) The emission spectrum when the injection current is set to be 200 mA. Reprinted with permission from [122] © Optical Society of America.

In addition to active/passive photonic chips, there is a growing interest in compound TFLN PIC designs. These designs are being proposed and studied to meet the diverse and expanding demands for on-chip microslaser applications. In order to develop the urgently needed integrated optical transmitters for wavelength division multiplexing (WDM), Y. Han, et al. have demonstrated an electro-optic tunable O-band hybrid TFLN/III-V laser as the schematic illustrated in Figure 10a [122]. The TFLN PIC comprises two cascaded thermo-optic (TO) tunable microrings, commonly referred to as the Vernier filter, along with a DBR, as shown in Figure 10b. The DBR and the partially reflective (PR) coating on the rear facet of the InP RSOA chip composed the FP cavity. The light–current (L–I) measurement is conducted and the recorded L–I curve is depicted in Figure 10c, showing the laser works in a continuous-wave mode with a threshold current of 100 mA. The maximum output power is 2.5 mW at 300 mA, which can be improved by optimizing the reflectivity of the partially reflective (PR) coating in the RSOA chip and the TFLN DBR. The fluctuations in the L–I curve evidences the existence of mode hopping in the hybrid laser cavity [123]. The central wavelength of the single-mode lasing peak is measured to be 1325.5 nm at 200 mA pump current, and the SMSR is higher than 60 dB as shown in Figure 10d. The on-chip wavelength tuning with a tuning range of 34 nm and a 0.42 nm/mW efficiency is demonstrated by adjusting the voltages applied on the integrated TO microheaters. The device can be employed to assemble a tunable O-band optical transmitter by directly coupling with a Mach–Zehnder modulator (MZM) with a capacitance-loaded traveling-wave electrode featuring a large EO bandwidth [88]. The optical transmitter support data rate is measured to be 160 Gb/s.

Y. Ren, et al. reported on a widely and quickly tunable ECSL supported by a TFLN chip composed of three cascaded EO tunable microrings, a PM, and a loop mirror (LM) [124]. The TFLN chip is butt-coupled to an RSOA with a highly reflective (HR) coating rear facet through a spot size converter (SSC). A shallow etch is conducted for improving the tuning efficiency of the single microring, which is critical for achieving a wide wavelength tuning range. The hybrid FP laser exhibits an ultra-wide lasing wavelength tuning range of 96 nm across C + L + U bands and a switch rate of 18 ns.

**Figure 11:**
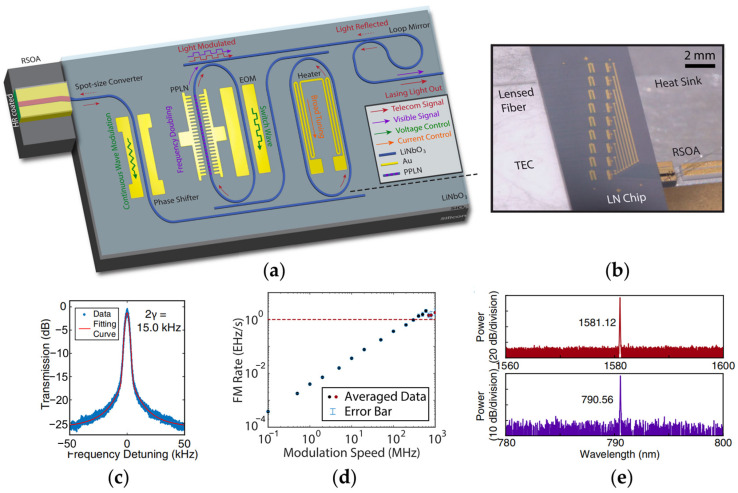
Tunable integrated Pockels laser based on the a TFLN photonic chip [125]. (**a**) Schematic of the device. (**b**) Optical microscope image of the integrated device. (**c**) Beating signal of laser recorded from a sub-coherence delayed self-heterodyne measurement. (**d**) The output lasing frequency modulation rate versus the EO modulation speed. (**e**) Optical spectra of the laser with two lasing wavelengths. Top inset: the spectrum of the fundamental frequency lasing. Bottom inset: the spectrum of the SHG lasing.

The strong optical nonlinearity of lithium niobate has offered a great opportunity for on-chip TFLN classic and quantum light sources. With the integration of optical gain provided by a III-V medium, on-chip electrically pumped multi-color lasers based on a PPLN racetrack ring are demonstrated by harnessing the large Pockels effect of LN, as mentioned in Section 2.1. The tunability and reconfigurability performance of the device can be further optimized and enriched by using a compound TFLN photonic chip, which includes a spot size converter, double-racetrack Vernier rings, a tunable phase controller, and a Sagnac loop ring, as illustrated in Figure 11a [125]. The racetrack rings of the Vernier mirror are incorporated with different TO, EO, and nonlinear optical components, which enables the device to support on-chip broad and fast wavelength tuning and the SHG process. Figure 11b is the digital picture of the device sitting on the heat sink. The beating signal recorded using the delayed self-heterodyne method is fitted by a combination of Lorentzian and Gaussian profiles, evidencing a linewidth of 15.0 kHz, as shown in Figure 11c. The highest laser frequency modulation rate achieves 2.0 EHz/s (2.0 × 10^18^ Hz/s) when the EO modulation speed is at 600 MHz as depicted in Figure 11d. The output spectrum of the laser is shown in Figure 11e, which confirms a double wavelength lasing of both the fundamental wave and the SHG wave. Fast on–off switching of the fundamental and SHG lasing modes is observed at a rate of 50 MHz and 10 MHz, respectively.

## 3. Conclusions and Future Perspective

**Figure 12:**
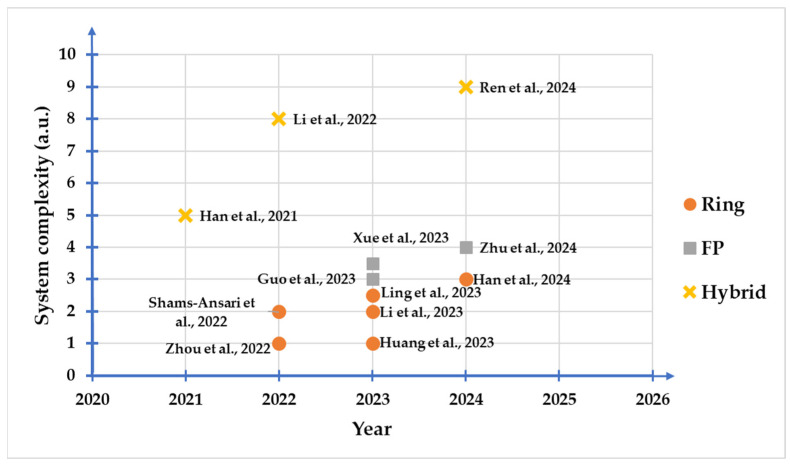
The evolution timeline of the integration density and configuration of the hybrid TFLN ECSL [86,87,111,112,113,114,115,118,119,122,124,125].

After nearly a decade of development, the advantages of thin film lithium niobate photonic devices in cutting-edge fields such as astronomical observation, artificial intelligence, microwave communication and high-speed communication have become increasingly prominent. Meanwhile, the demand for high-performance on-chip light sources is increasing day by day. Here, we review the recent progresses on the ECSLs based on the integration of compound semiconductor devices and TFLN photonic chips. Impressive enhancements have been achieved in critical parameters of hybrid lithium niobate external cavity semiconductor lasers, as shown in Table 2, including emission linewidth, frequency noise, insertion loss, and EO tuning functionalities. A brief roadmap in the advancements of the integration density is also provided in Figure 12. Each configuration of the ECSLs is becoming more complex with an increasing chip-scale.

It is worth mentioning that currently, the properties of these devices have not yet reached the physical limits of lithium niobate photonic devices. A series of tasks must still be accomplished. First, the mode field mismatch between the semiconductor diode or gain chips and the LN waveguides impedes the suppression of the coupling loss. The thickness of the thin film lithium niobate and the design of spot size converter should be taken into careful consideration. Secondly, the practical applications of current TFLN ECSL devices are hindered by their relatively low output power, typically ranging from a few hundred microwatts to several tens of milliwatts. As the uniformity of TFLN is crucial for fabricating high quality photonic structures and restraining the on-chip power consumption, it is necessary to further reduce the defects that may be induced during the ion-slicing or waveguide-etching processes. It is worth noting that magnesium-doped lithium niobate often has a higher damage threshold and resistance to photorefractive effects, which is an ideal platform for achieving high-power laser application. In addition, the superior nonlinear optical properties and EO tuning rate of lithium niobate have not been fully utilized.

The stability of the ECSLs has been a topic of significant interest. It has been characterized in only a few hybrid TFLN ECSLs via measuring of the frequency noise, also referred to as phase noise, which generally refers to the random fluctuations in the frequency or phase of the output signal caused by various noise sources within the system. The frequency noise of a single-frequency laser is typically characterized by the spectral density of frequency fluctuations in the frequency domain, emphasizing the frequency jitter over short time intervals. From the reported frequency noise data, it can be observed that the FP cavity [119] exhibits lower frequency noise compared to the microring cavity [113,114], indicating better short-term stability. Meanwhile, the long-term stability of frequency in the time domain is also valuable for the applications of narrow-linewidth lasers. Hybrid integrated frequency-stabilized lasers have already achieved in packages on platforms such as silicon [126,127] and silicon nitride [128,129], but similar studies on the lithium niobate platform is still in their infancy. It is noteworthy that very recently an on-chip lithium niobate micro-disk laser based on a hydrogen cyanide (H_13_C_14_N) gas saturation absorption method for frequency stabilization has been demonstrated [130]. By operating on the H_13_C_14_N P12 absorption line at 1551.3 nm, the laser frequency can be precisely stabilized with a best stability value of 9 × 10^−9^. The short-term stability, evaluated over continuous time intervals of 35 s, showcases exceptional performance. The residual drift remains well below 30 MHz. The frequency stability can be further improved using the on-chip EO feedback scheme, which can benefit precision metrology, and high sensitivity sensing. In addition, the stability of the laser frequency is affected by many factors, such as changes in refractive index due to temperature variations, mode hops, and alterations in cavity length, all of which can lead to laser frequency drift. Furthermore, the lithium niobate lasers reported so far have all employed spatial coupling methods, making the laser frequencies more susceptible to fluctuations in the external environment compared to packaged lasers.

Hence, there is still significant room for development in TFLN integrated photonics technology, indicating its immense potential in high-speed optical communication, LiDAR, quantum information and artificial intelligence technology.

## Figures and Tables

**Table 1:** The measured loaded Q factors of 12 microring samples with bus waveguides prepared in the same PLACE batch without annealing.

Sample No.	Wavelength (nm)	Loaded Q (×10^6^)
1	1562.74	1.53
2	1566.53	1.87
3	1559.77	0.84
4	1553.87	1.39
5	1568.44	1.64
6	1556.05	1.15
7	1559.03	2.82
8	1549.55	2.92
9	1568.89	2.25
10	1555.90	3.27
11	1567.73	2.59
12	1553.50	3.06

**Table 2:** Summary of the hybrid TFLN/semiconductor lasers reported in the literature.

Main Structures of the TFLN Photonic Chip	Semiconductor Chip	Wavelength	Output Power	Linewidth	Coupling Loss	Tuning Range (Method)	SMSR	Ref.
Er^3+^ doped MRR	GaAs/AlGaAs DFB laser	1531.27 nm	500 nW	0.05 nm	10 dB	/	/	[111]
MRR	GaAs/AlGaAs DFB laser	982 nm	4.27 mW	2 nm	10 dB	2.57 nm (DFB current)	/	[112]
MRR array	InP DFB laser	1558 nm	19.8 mW	<1 MHz	10 dB	/	/	[87]
racetrack MRR	InP DFB laser	1556 nm	~0.4 mW	~242 kHz	/	760 MHz (EO)	60 dB	[113]
PPLN racetrack MRR	InP DFB laser	779.8 nm (SHG)	2 mW (on-chip)	4.7 kHz	/	/	/	[114]
PM + racetrack MRR	DFB laser	1550 nm	3.18 mW	2.5 kHz	6 dB	/	60 dB	[115]
MZI + SLR	InP ROA	1544 nm	738.8 μW	45.5 kHz	/	0.16 nm, 20 GHz (EO)	/	[118]
PM + broadband LM	Single-angled facet (SAF) GaAs gain chip	1065 nm	50 mW (average)	0.35 nm	3.4–3.9 dB	/	/	[86]
PS + DBR	RSOA	1516.21 nm	2.1 mW	94 kHz	/	8 GHz (EO)	50 dB	[119]
two cascaded MRRs + DBR	InP RSOA	1331.88 nm	2.5 mW	/	/	36.4 nm (TO)	60 dB	[122]
PS + 3 cascaded MRRs + LM	InP RSOA	1614.3 nm	0.98 mW	/	1.87 dB	96 nm (EO)	52 dB	[124]
PS + 2 cascaded racetrack MRRs + LM	RSOA	1581.12 nm	5.5 mW (on-chip)	11.3 kHz	3–4 dB	20 nm (TO); 1.2 GHz (EO)	50 dB	[125]

## Data Availability

Data are contained within the article.

## Abbreviations

DBR	distributed Bragg reflector
DC	directional coupler
DFB	distributed-feedback
EBL	electron beam lithography
ECDL	external cavity diode laser
ECSL	external cavity semiconductor laser
EO	electro-optic
FP	Fabry–Pérot
FWHM	full width at half maximum
GSG	ground–signal–ground
LD	laser diode
LM	loop mirror
LiDAR	light detection and ranging
MLL	mode-locked laser
MRR	micro-ring resonator
MZI	Mach–Zehnder interferometer
MZM	Mach–Zehnder modulator
PIC	photonic integrated circuit
PLACE	photolithography-assisted chemo-mechanical etching
PM	phase modulator
PS	phase shifter
PPLN	periodically poled lithium niobate
REI	rare-earth ion
RF	radio frequency
RSOA	reflective semiconductor optical amplifier
SAF	single-angled facet
SEM	scanning electron microscope
SHG	second-harmonic generation
SLR	Sagnac loop reflector
SMSR	side mode suppression ratio
SSC	spot size converter
SOA	semiconductor optical amplifier
TFLN	thin film lithium niobate
TO	thermo-optic
UV	ultraviolet
